# Metabolic Features and Nutritional Interventions in Chronic Diseases—2nd Edition

**DOI:** 10.3390/nu18152485

**Published:** 2026-08-01

**Authors:** Junjie Luo, Peng An, Yongting Luo

**Affiliations:** Department of Nutrition and Health, China Agricultural University, Beijing 100193, China; luojj@cau.edu.cn

Metabolic dysfunction is increasingly recognized as a central driver in the pathogenesis and progression of a wide spectrum of chronic diseases, ranging from age-related disorders and metabolic syndrome to cardiovascular diseases, cancer, and gastrointestinal pathologies [1,2,3]. Nutritional interventions, whether through the application of bioactive compounds, probiotics, prebiotics, or tailored dietary regimens, have been shown to effectively counter these metabolic disturbances, helping to mitigate disease progression and improve clinical outcomes [4]. This Special Issue of *Nutrients*, entitled “*Metabolic Features and Nutritional Interventions in Chronic Diseases—2nd Edition*”, aims to bring together recent work on the metabolic features of chronic diseases and to explore how nutritional interventions can be used to regulate metabolism and improve disease management.

This Special Issue comprises seven original research articles and one systematic review, covering a diverse range of topics that underscore the broad therapeutic potential of nutritional strategies. The topics covered include the following: the anti-sarcopenic effects of α-ketoglutaric acid (AKG) in D-galactose-induced aging mice via modulation of protein homeostasis and optimization of mitochondrial function (Contribution 1); the amelioration of sarcopenia by broccoli-derived peptides (BDP) and leucine in combination through multimodal regulation of multiple signaling pathways (Contribution 2); the synergistic anti-fatigue effects of whey peptides combined with micronutrients in exercise-trained mice (Contribution 3); the enhancement of intestinal amino acid absorption by *Lactobacillus paracasei* L9 in aged mice through the butyric acid-Akt/mTOR-LAT2 axis (Contribution 4); the protective effects of toddler-derived fecal microbiota transplantation (FMT) against dextran sulfate sodium (DSS)-induced colitis in mice (Contribution 5); the synergistic effects of mulberry leaf and corn silk extracts combined with milk matrices on postprandial glucose regulation in prediabetic individuals (Contribution 6); the therapeutic potential of zinc-enriched *Bifidobacterium longum* subsp. *longum* CCFM1195 in alleviating acne through anti-inflammatory and antioxidant mechanisms (Contribution 7); and a systematic review evaluating the association between dietary patterns, dietary interventions, and sinonasal outcomes in rhinosinusitis (Contribution 8).

Aging is accompanied by a progressive loss of muscle mass and strength, driven by oxidative stress, mitochondrial dysfunction, and an imbalance between protein synthesis and degradation [5,6]. Similarly, strenuous exercise generates excessive reactive oxygen species and inflammatory responses that impair muscle recovery and performance [7]. Nutritional interventions that target these pathways, whether through amino acid derivatives, bioactive peptides, or micronutrient combinations, have emerged as promising strategies to preserve muscle function. First, three papers in this Special Issue address skeletal muscle deterioration and physical fatigue. Zhang Y. et al. show that AKG counteracts sarcopenia by rebalancing protein synthesis/degradation through the Akt/mTOR axis and by enhancing mitochondrial antioxidant defence via activating the SIRT1/PGC-1α/Nrf2 pathway (Contribution 1). Complementing this, Yuan K. et al. demonstrate that BDP and leucine, particularly in combination, exert superior anti-sarcopenic effects: BDP enriches actin-cytoskeleton and anti-inflammatory pathways, leucine activates Apelin and ECM signalling, and their co-administration uniquely engages MAPK signalling to promote myogenic differentiation while suppressing catabolic and inflammatory mediators (Contribution 2). Beyond chronic muscle loss, Cheng Y. et al. tackle acute exercise-induced fatigue and show that whey peptides combined with micronutrients synergistically increase muscle mass, muscle fiber cross-sectional area, lower serum fatigue biomarkers (blood urea nitrogen, lactate acid, and creatine kinase), and attenuate excessive immune activation in muscle, thereby improving both endurance and strength (Contribution 3).

The gut microbiota is a metabolic hub that regulates energy, immunity, and nutrient bioavailability [8,9]. Age-related dysbiosis impairs barrier function and reduces short-chain fatty acid production, compromising amino acid absorption and promoting inflammation [10,11]. Microbiota-targeted interventions such as probiotics and FMT have thus gained attention. The next two studies examine the gut ecosystem’s role in amino acid supply and intestinal health. Li W. et al. reveal that long-term *Lactobacillus paracasei* L9 supplementation in aged mice reshapes the gut microbiota, elevating butyric acid production (Contribution 4). This metabolite then activates the Akt/mTOR pathway and upregulates the neutral amino transporter LAT2 in the intestine, leading to increased plasma amino acid levels and improved protein digestibility. In parallel, Jing Y. et al. employ toddler-derived FMT in a DSS-induced colitis model (Contribution 5). FMT enriches beneficial genera (*Bacteroides*, *Parabacteroides*, *Blautia*, and *Akkermansia*), restores goblet cell density and MUC2 expression, reduces CD4^+^ T-cell infiltration, and downregulates JAK/STAT signaling—effectively ameliorating colonic inflammation and barrier disruption, which are key to maintaining systemic metabolic homeostasis.

Chronic low-grade inflammation and oxidative stress are common pathogenic threads linking diverse metabolic diseases [12,13], from hyperglycemia and insulin resistance to skin inflammation and upper airway disorders. Dietary components, whether as whole foods, bioactive extracts, or probiotic formulations, can modulate these processes by interfering with digestive enzymes, regulating immune signaling cascades, or reshaping the systemic inflammatory milieu. Beyond the gut, this theme unites the final three studies. Sun Y. et al. combine mulberry leaf and corn silk extracts, which synergistically inhibit α-amylase and α-glucosidase in vitro with milk matrices (Contribution 6). In a randomized crossover trial in prediabetic individuals, the combination of GOS milk supplemented with mulberry leaf and corn silk significantly reduces 1 h postprandial glucose and glucose excursion in overweight participants, offering a practical nutritional strategy for glycemic control. For inflammatory skin conditions, Gu X. et al. develop a zinc-enriched *Bifidobacterium longum* subsp. *longum* CCFM1195 and test it in a zinc-deficient acne mouse model (Contribution 7). This probiotic outperforms inorganic zinc sulfate in reducing lesion size, suppressing IL-1β, IL-17A, and MDA, and enhancing GSH-Px activity, while downregulating TLR2/NF-κB signaling—illustrating how targeted micronutrient–probiotic combinations modulate both innate immunity and redox balance. Finally, a systematic review by Martinez C. et al. synthesizes ten studies on dietary patterns and rhinosinusitis, concluding that anti-inflammatory diets (rich in fruits, low in ultra-processed foods) are consistently associated with lower disease prevalence and symptom severity, whereas Western dietary patterns correlate with worse outcomes (Contribution 8). This reinforces the concept that overall dietary quality, through its impact on systemic inflammation and oxidative stress, is a modifiable determinant of chronic sinonasal disease.

In summary, the studies in this Special Issue collectively underscore the therapeutic potential of nutritional strategies in modulating metabolic pathways, oxidative stress, immune responses, and gut microbiota composition across a diverse range of chronic diseases. From aging-related muscle decline and exercise-induced fatigue, through intestinal dysbiosis and colitis, to postprandial hyperglycemia, inflammatory skin conditions, and chronic sinonasal inflammation, these findings reinforce the paradigm of “food as medicine” [14,15] and highlight the need for further clinical exploration of personalized nutritional interventions. The convergence of evidence from molecular mechanisms, preclinical models, and human studies attests to the concept that specific nutrients, probiotics, and dietary patterns can serve as effective adjuncts to conventional disease management. Future research should focus on elucidating the causal relationships between dietary factors and disease outcomes, identifying optimal intervention strategies for specific patient populations, and translating these findings into clinical practice.
